# Nanostructured Titanium Dioxide Surfaces for Electrochemical Biosensing

**DOI:** 10.3390/s21186167

**Published:** 2021-09-14

**Authors:** Linda Bertel, David A. Miranda, José Miguel García-Martín

**Affiliations:** 1 CMN-CIMBIOS Group, Escuela de Física, Universidad Industrial de Santander, Cra 27 Cll 9, Bucaramanga 680002, Colombia; linda.bertel@correo.uis.edu.co (L.B.); dalemir@uis.edu.co (D.A.M.); 2 Instituto de Micro y Nanotecnología, IMN-CNM, CSIC (CEI UAM+CSIC), Isaac Newton 8, E-28760 Madrid, Spain

**Keywords:** electrochemical biosensors, nanostructured surfaces, titanium dioxide, functionalization with biomolecules

## Abstract

TiO_2_ electrochemical biosensors represent an option for biomolecules recognition associated with diseases, food or environmental contaminants, drug interactions and related topics. The relevance of TiO_2_ biosensors is due to the high selectivity and sensitivity that can be achieved. The development of electrochemical biosensors based on nanostructured TiO_2_ surfaces requires knowing the signal extracted from them and its relationship with the properties of the transducer, such as the crystalline phase, the roughness and the morphology of the TiO_2_ nanostructures. Using relevant literature published in the last decade, an overview of TiO_2_ based biosensors is here provided. First, the principal fabrication methods of nanostructured TiO_2_ surfaces are presented and their properties are briefly described. Secondly, the different detection techniques and representative examples of their applications are provided. Finally, the functionalization strategies with biomolecules are discussed. This work could contribute as a reference for the design of electrochemical biosensors based on nanostructured TiO_2_ surfaces, considering the detection technique and the experimental electrochemical conditions needed for a specific analyte.

## 1. Introduction

The use of biosensors covers several areas of knowledge, such as biomedical research, forensic investigation, drug discovery, point-of-care diagnostics, environmental monitoring and food control [1]. Biosensors allow the selective detection of analytes, taking advantage of the affinity interaction they present with specific bioreceptors immobilized on the surface of the sensor [2], e.g., enzyme-substrate, DNA-target DNA or antibody-antigen interactions [3].

To prepare biosensors, different types of transducers can be implemented to convert the biorecognition event into a measurable signal as electrochemical, optical or other physical variables [1,2,3,4,5,6]. Electrochemical transducers are widely used in biosensor devices due to the diversity of experimental electrochemical setups, simple data collection and robust interpretation.

Nanoscale materials (nanoparticles, nanotubes, nanowires and nanosheets) have been widely implemented in the design of high-performance electrochemical biosensors due to their high surface-to-volume ratio and excellent electrical properties. Among them, carbon nanotubes (CNTs) have been a preferential object of study since their discovery in the 1990s as a base material for the development of promising electrochemical biosensors due to their superior resistance [7]. Furthermore, CNTs also exhibit excellent conductivity, high sensitivity, good biocompatibility, exceptional chemical stability and easy functionalization with almost any desired chemical species [8]. Sensors based on nanostructured metal oxides of metals such as Cu, Zn, Ni, Fe and Ti have advantages as lower limits of detection, wide linear range, reproducibility and stability. CuO-based electrochemical biosensors have been developed for glucose detection with good stability and sensitivity [9,10,11,12]. The main advantages of using CuO nanomaterials are precise surface area, good electrochemical activity and the ability to stimulate electron transfer reactions at lower potentials. ZnO nanostructured materials have interesting properties such as high electrical conductivity, nontoxicity, cost-effectiveness and chemical steadiness [9,13]. Moreover, it has been found that in glucose biosensors, the sensitivity is improved using zinc oxide nanocomposites linked to simple graphene. Nanostructured NiO is also an ideal choice for electrochemical biosensors and immobilization of biomolecules owing to its high isoelectric point, porous morphology, smaller crystallite size, biocompatibility, chemical stability and high surface redox activity, in addition to being preferred due to the low cost of its metal oxide constituents. Electrochemical glucose sensors based on graphene nanocomposites constructed with nickel oxide exhibit excellent electrocatalytic properties, reasonable compatibility and the absence of interference through additional electroactive types such as ascorbic acid and dopamine, and uric acid [9,14].

This work is focused on electrochemical biosensors using nanostructured TiO_2_ surfaces (NTOS) as working electrode. These NTOS can be thin films with tailored roughness or surface composed of arrays of nanostructures. They are of particular interest in developing electrochemical biosensors because they have a more significant number of optimal properties for this application, in comparison to other materials that are also used as sensors, such as biocompatibility, non-toxicity, corrosion resistance, low fabrication costs, transduction mechanisms, high surface area, quantum confinement, efficient electronic charge properties and high adhesion to the substrate [15,16,17]. It is even known that monolayers of nanoparticles in the anatase phase show good electronic connectivity with the substrate, which allows TiO_2_ nanoparticle layers to be used as electrodes [18].

Another factor that influences the selection of NTOS for biosensing applications is that the electrochemistry of TiO_2_ has been a widely studied area since its birth in 1969. This extensive knowledge of the electrochemistry of TiO_2_ is a key point to understand, improve and optimize the phenomena involved in the sensing process (charge generation, injection, separation, transport or accumulation in the nanostructure) [18]. In NTOS-based electrochemical biosensors, electrons from the reaction between the bioreceptor and analyte can be collected by the TiO_2_ thanks to its electron-accepting character; they are consequently used to detect the reaction [15,16]. Moreover, it should be noted that these NTOS are excellent candidates for the assembly of different types of biorecognition molecules such as antibodies, enzymes or receptors [4].

For electrochemical biosensors based on TiO_2_, a key important characteristic is its crystalline phase, in clear contrast to energy storage applications in which the main parameter is the particle size [19]. The more common crystalline phases of TiO_2_ are anatase (tetragonal), brookite (orthorhombic) and rutile (tetragonal). Rutile is generally the thermodynamically most stable bulk phase at most temperatures and pressures. However, in many cases, when TiO_2_ films are prepared, they turn out to be amorphous, requiring an additional annealing process to achieve a phase transformation. A transformation from amorphous to anatase structure is obtained by annealing at temperatures of 300–500 °C, and a transformation from anatase to rutile phase, at 600–700 °C [15,19]. The performance of TiO_2_ in many applications depends on its crystallinity. In photocatalytic applications, it has been found that a mixture of anatase and rutile phases is a very efficient configuration since photo-excited electrons and holes are trapped, inhibiting the electron-hole recombination that significantly limits photocatalytic efficiency [20]. In electrochemical biosensors, the use of a thin film of TiO_2_ in the anatase phase is desirable because of its insolubility against moderate acid and alkali solutions. This property makes the TiO_2_ anatase phase especially suitable for the development of pH sensors [21]. Moreover, although many applications are performed in physiological pH, the previous step of functionalization with different biomolecules may involve more restrictive conditions under acidic or basic pH. The anatase phase of TiO_2_ allows the NTOS, of many electrochemical biosensors, to remain stable during such functionalization processes. As the fabrication method determines the type of NTOS and the crystalline phases obtained, Section 2 is devoted to material preparation.

Review articles such as that of Berger et al. show how the electrochemical properties of electrodes based on TiO_2_ nanostructures determine their application, whether as supercapacitors, electrochromic layers and photocatalysts in the degradation of organic pollutants [18]. Shetti et al. show recent achievements in biosensors based on hybrid TiO_2_ nanostructures, emphasizing examples of photoelectrochemical, electrochemiluminescence, amperometric, conductometric, enzyme-based, doped TiO_2_ nanoparticles and TiO_2_-graphene hydrides-based biosensors [22]. Furthermore, Nadzirah et al. focused on showing applications of electrical biosensors based on TiO_2_/organic nano-hybrid and inorganic/TiO_2_ nano-hybrid, especially for field-effect transistor and interdigitated electrode (IDE) detections [23].

In Section 3, we have selected relevant literature from the last decade, focusing our attention on the different electrochemical detection techniques that can be used and pinpointing the key factors in each case: voltammetric/amperometric, potentiometric, conductometric and impedimetric detectors, and Field Effect Transistor biosensors (BioFETs). Furthermore, as another novelty compared to the review articles mentioned above, Section 4 is devoted to discussing the functionalization of NTOS, including the possibility to modify the stability, reactivity and functionality chemical surface properties of these NTOS by using some functional groups, e.g., silane, carboxylate and amine. The use of biomolecules is another way to modify the NTOS properties. It can be carried out by physical or chemical interaction, physical interactions (as hydrophobic interactions, hydrogen bridges and electrostatic interactions) and chemical (as chemical bonding strategies such as covalent coupling) [24,25]. The NTOS functionalized with biomolecules allows for the selective recognition of an analyte.

## 2. Preparation of TiO_2_ Nanostructured Surfaces

This section shows how a specific manufacturing process can determine the shape and crystalline phase of nanostructured TiO_2_ surfaces. In addition, experimental details are provided that can facilitate the new researcher to get a broad idea about the manufacturing method.

### 2.1. Electrochemical Anodization

**Figure 1:**
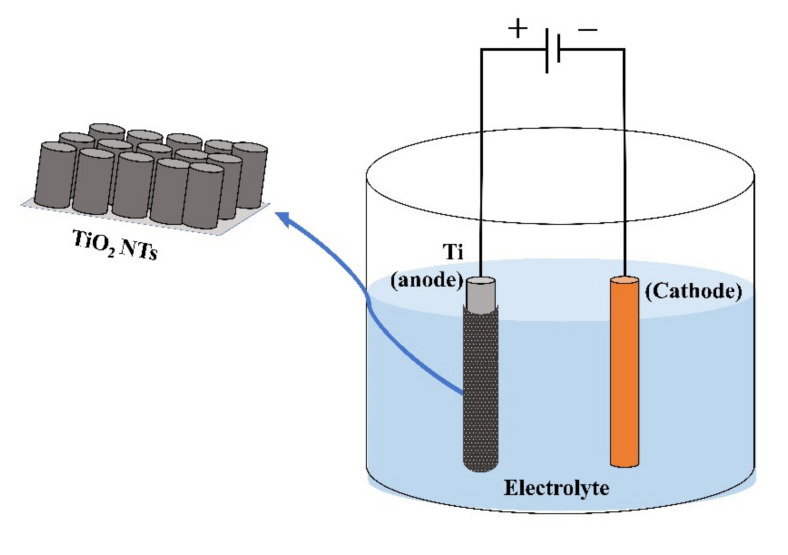
The electrochemical anodization process for obtaining TiO_2_ NTs.

Anodization is a widely used technique for obtaining organized TiO_2_ nanotubes (NTs) since it allows excellent control of growth parameters (potential, time, temperature and type of electrolyte) [26]. In a general way, TiO_2_ NTs (with anatase phase) are fabricated by the two-step electrochemical anodization of a Ti metal foil in an ammonium fluoride (NH_4_F)-ethylene glycol solution (see Figure 1). Ti foil served as anode and a graphite or Platinum foil or wire as cathode, applying electrical potential at room temperature (RT). Finally, the anodized samples were subjected to an annealing process at 350–500 °C and a constant heating rate [26,27]. After the fabrication process, biosensing application of NTs usually implies their immersion in aqueous solution, where structural changes could be presented and affect its stability. In this sense, Cao et al. fabricated TiO_2_ NTs arrays by electrochemical anodization and studied their stability in distilled water and in a cell culture media with minimum essential medium (α-MEM) supplemented with 15% fetal bovine serum (FBS) and PBS [28]. They found that after 3 h of immersion in distilled water at 37 °C, the NTs that had an annealing stage after their fabrication showed good stability, while the NTs that did not have the annealing stage experienced a modification in their morphology with amorphous TiO_2_ dissolution and anatase TiO_2_ recrystallization. However, the non-annealed NTs showed stability in the culture media without evident changes in their morphology and phase; this could be ascribed to the presence of inorganic species that can prevent the thermodynamic processes of amorphous TiO_2_ dissolution and anatase TiO_2_ recrystallization. Other factors that can increase structural change events are temperature and the presence of F- (generated in the manufacturing process of TiO_2_ NTs by electrochemical anodization).

Other interesting fabrication process was reported by Gualdrón-Reyes et al., who implemented an anodization method to obtain boron, nitrogen or fluorine-tridoped TiO_2_ NTs membranes, which were later adhered onto indium-tin-oxide (ITO) conductive glass and sensitized with different CdSe quantum dots loading using the successive ionic layer adsorption and reaction (SILAR) method. The SILAR deposition technique consists of a cycle of immersion of the TiO_2_ NTs substrate in solutions of cationic and anionic precursors (3CdSO_4_8H_2_O and SeO_2_), washing and reaction. This surface was fabricated to study their photochemical, photoelectrochemical and semiconducting properties [29]. Even though the anodizing method typically produces NTs from Ti, it has been found that the TiO_2_ geometry can be modified to obtain tube stacks, bamboo and double-walled and amphiphilic double-layer tubes, through variations in the anodizing voltage, duration and composition of the electrolyte solution [15].

### 2.2. Sol-Gel

**Figure 2:**
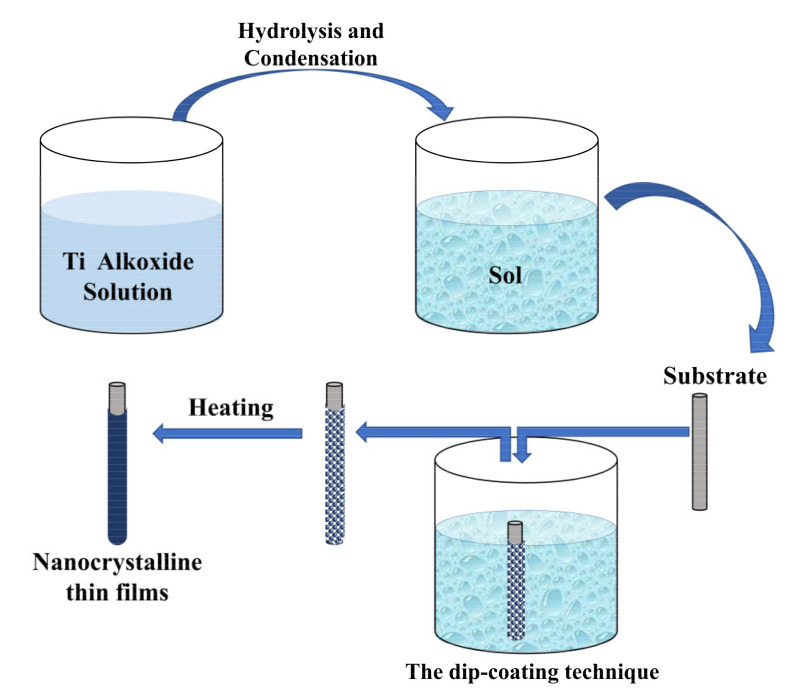
Sol-gel process for obtaining TiO_2_ thin films. The process begins with the formation of the sol by hydrolysis and condensation of titanium alkoxides mixed with alcohol and catalytic agents. After the deposition, by dip-coating, the film is formed. Next, a thermal treatment is used for the preparation of nanocrystalline thin films.

The sol-gel process allows obtaining nanostructured thin films following the steps shown in Figure 2 and using the dip-coating technique (which involves immersing a substrate in the sol and retiring it at a constant speed) [30]. Chen et al. developed a urea biosensor based on the immobilization of urease onto a TiO_2_ nanoporous film. A mixture of urease and TiO_2_ gel was subjected to ultrasonic radiation. Titanium wires were immersed in the above solution for 1 min, removed (from the solution), and dried in air. They were washed with a NaOH solution. This film was then subjected to a sintering process at 300 °C to give the film stability. XRD patterns of obtained TiO_2_ film indicate the TiO_2_ phase is anatase [31].

NTOS with other geometry, such as nanotube arrays with nanopores on their walls, have been fabricated using the double-template-assisted sol-gel method, where the TiO_2_ coating has the anatase crystalline phase [32].

### 2.3. Hydrothermal Method

**Figure 3:**
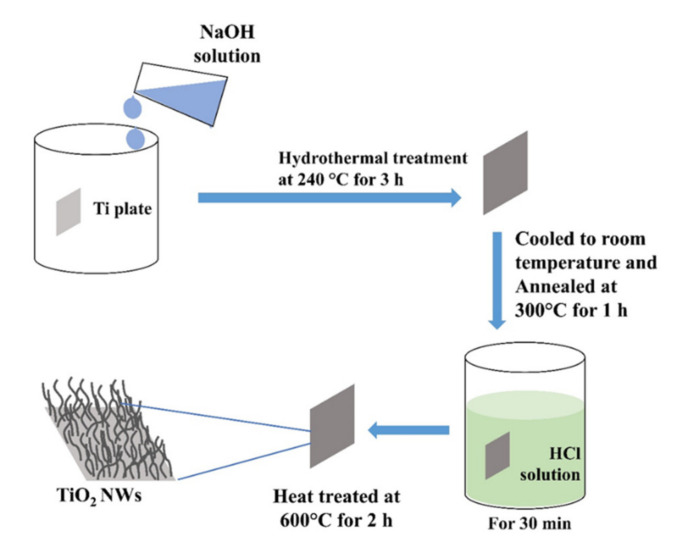
Hydrothermal method for obtaining TiO_2_ NWs.

TiO_2_ nanowires (NWs) were fabricated by the hydrothermal method (see process overview in Figure 3) onto the surface of titanium plates (1 cm^2^) in NaOH, varying some reaction condition (NaOH concentration [1 or 2 M], hydrothermal reaction temperature [120 or 240 °C], and reaction time [1 or 3 h]) [33]. Samples obtained from the hydrothermal reaction were cooled down to RT, annealed at 300 °C and submerged in HCl solution to form TiO_2_. These samples were subjected to a final calcination process at 600 °C. XRD and RAMAN analysis confirm the presence of anatase and rutile phases in all samples. However, the weight percentage of crystalline phases is dependent on the reaction time and temperature: at lower temperature and shorter reaction time, high relative weight percentages of rutile are generated and at the higher reaction temperature (240 °C), anatase is the majority phase. Moreover, the main parameter that allows the modification of the surface morphology is the NaOH concentration. An interconnected NWs frame is formed by increasing the concentration, with considerable lengths compared to the individual NWs observed in other samples prepared with lower concentration.

Wu-Qiang et al. developed a novel and simple one-step hydrothermal reaction to obtain long TiO_2_ NWs coated with short TiO_2_ nanorods (NRs), with anatase phase, on fluorine-doped tin oxide (FTO) glass using a hydrothermal reaction of K_2_TiO(C_2_O_4_)_2_ in water and diethylene glycol [34].

TiO_2_ NRs can also be prepared on a Ti substrate through a hydrothermal process. Initially, the Ti substrate was deposited in a hydrothermal solution consisting of a mixture of ethanol, H_2_O, HCl and titanium tetrabutoxide, subjected to a temperature of 160 °C. After cooling the samples and washing them with deionized water and ethanol, rutile phase NRs were obtained. In a second stage, these NRs were hydrothermally alkalinized using a solution of hydroxides (KOH/NaOH = 1:1) at 200 °C, washed with deionized water and immersed in a 0.1 M HCl solution. Finally, the NRs were heated in an oven at 500 °C to obtain anatase TiO_2_ NRs. These NRs over Ti electrodes were submitted to an electrochemical reduction process to obtain Ti^3+^-TiO_2_NR/Ti [35].

### 2.4. Spray Pyrolysis

**Figure 4:**
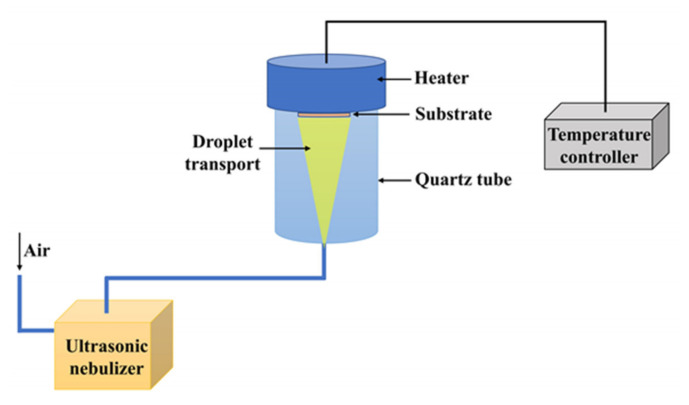
SP configuration for obtaining TiO_2_ thin films. A static ultrasonic nebulizer generates a uniform distribution of the aerosols in the diameter range of 1 to 3 µm. The aerosols generated are transported to the heated substrate (between 300 and 550 °C), evaporate and react (on the substrate) to form the TiO_2_ films.

Spray Pyrolysis (SP) is commonly used to synthesize solid powders and films of metal oxide. However, metals and semiconductors can also be obtained by making a convenient choice of deposition environment and carrier gas, i.e., by using the appropriate composition of the precursor solution, the suitable carrier gas and the flux rate that facilitate the reactive interaction with such precursor [36,37]. Raut et al. obtained thin films of nanocrystalline TiO_2_ on glass and quartz substrates by SP, using the configuration shown in Figure 4. In this process, the following conditions were used: titanium-oxy-acetyl acetone in ethanol as a precursor solution (0.01–0.1 M), a static ultrasonic nebulizer generates aerosols, air as a carrier gas and heated substrates at 300–550 °C. A correct adjustment of the deposition conditions (precursor concentration, the flow rate of solution, the flow rate of air, atomization rate, substrate temperature and deposition time) was necessary to produce TiO_2_ films with an anatase crystalline phase and ∼20 nm nominal grain sizes determined from high-resolution SEM (HRSEM) studies [36].

### 2.5. Atomic Layer Deposition

**Figure 5:**
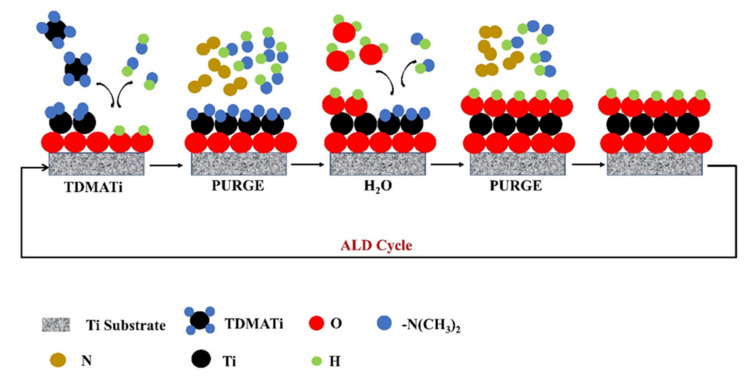
Scheme showing the preparation of TiO_2_ thin films using ALD. TDMATi reacts with the –OH terminated surface of the Ti substrates (with TiO_2_ superficial), leaving a methyl terminated surface. N_2_ subsequently cleans the surface of compounds not adhering to the substrate. Water later reacts with the methyl groups, creating a surface with –OH groups. Next, N_2_ cleans the surface again. This process is repeated over several cycles to obtain a film with the desired thickness.

Atomic Layer Deposition (ALD) is a technique that allows to obtain controlled coatings by superficial reactions in different steps (see a proposed reaction scheme for TiO_2_ ALD on Ti substrate in Figure 5). This technique is widely used to make deposits of metal oxide layers. In the deposition of TiO_2_ by ALD, titanium tetrachloride, titanium isopropoxide and tetrakis(dimethylamino)titanium (TDMATi) have been mainly used as precursors, and water or ozone have been used as an oxidant agent. Liu et al. employed TDMATi and H_2_O for TiO_2_ thin film deposition on Ti substrates, and N_2_ was used as a purging gas. Steps of surface reactions (ALD cycle) were needed: 0.1 s in the presence of TDMATi, 6 s in N_2_, 0.1 s in water and 6 s in N_2_. ALD process employed 2500 cycles (to obtain ∼100 nm of the TiO_2_ film) for deposition temperatures of 120, 160 and 190 °C. AFM analysis showed a roughness of 40 nm for this NTOS, and XRD indicated the presence of crystalline anatase TiO_2_ films for samples prepared at 190 °C, in contrast with the samples deposited at 120 and 160 °C [38].

### 2.6. Sputtering

Magnetron sputtering allows the deposition of atoms with high uniformity and good adhesion to the substrate. When the target and the substrate are parallel, flat thin films are obtained, whereas if Oblique Angle Deposition (OAD) is used, corrugated films or even nanocolumns can be obtained depending on the tilt angle and the sputtering conditions. Comert et al. prepared TiO_2_ thin film onto an n-Si substrate by radio frequency (RF) magnetron sputtering technique, using a TiO_2_ target, directed with 45° deposition tilt angle, 2 × 10^−6^ Torr base pressure, 150 W RF power and 100 °C temperature of the substrate. Lastly, this film was annealed in the air from 500 to 1000 °C [39]. XRD analysis allowed to determine that for annealing temperatures ≤ 600 °C, the films remain in the anatase phase (present in the films deposited at 100 °C); at 700 °C, a mixture of anatase and rutile is evident, while at higher temperatures (≤ 800 °C) films with a structure in the rutile phase are obtained. In addition, it was observed by AFM analysis that an increase in annealing temperature led to an increase in surface roughness (the root mean square values of the surface roughness evolved from 0.2 to 7.4 nm), associated with an increase in grain size (from 13.20 to 122.50 nm), where the shape of the grains changed from columnar to cloudy.

**Figure 6:**
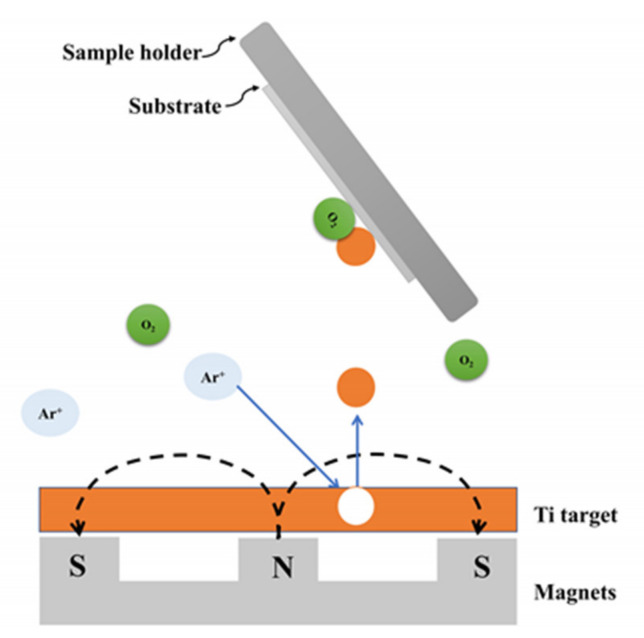
Schematic representation of the reactive sputter deposition process with OAD configuration. Oxygen atoms, originating from O_2_ gas molecules, can react with sputtered Ti atoms on the substrate to form a TiO_2_ film. The magnetic field (generated by the magnets) produces a force on the electrons, which keeps them on a helical path close to the target for relatively long times. In this way, very low sputtering gas pressures can be used together with an OAD configuration to allow the ejection of atoms to reach the substrate in an oblique direction.

OAD of TiO_2_ by reactive DC magnetron sputtering yields porous and amorphous TiO_2_ films with a columnar structure, using a Ti target, 385 W power, 0.02 Ar/O_2_ gas ratio, 12 mTorr working pressure and 60° tilt angle between substrate and target (Figure 6). After obtaining the film by sputtering, it was subjected to an annealing process in the air at 450 °C to achieve the amorphous to anatase transition [40]. However, Hu et al. achieved to fabricate separated TiO_2_ Nanocolumns (NCs) onto the compact TiO_2_-coated fluorine-doped tin oxide (FTO) substrates by a two-step process: (i) oblique deposition of Ti nanocolumn arrays using DC magnetron sputtering, and (ii) subsequent thermal oxidation of the Ti nanocolumns to transform them into TiO_2_ ones. The oblique deposition took place with a tilt angle between substrate and target higher than 60°, in particular 75°, using argon as sputter gas, 0.15 Pa pressure and 300 W power (DC discharge). Then, the Ti NCs were subjected to a thermal oxidation process (500 °C) to finally obtain the TiO_2_ NCs with an average diameter and length of 89 ± 18 nm and 254 ± 27 nm, respectively. These NCs present mainly the TiO_2_ rutile phase [41].

### 2.7. Electron-Beam Physical Vapor Deposition

**Figure 7:**
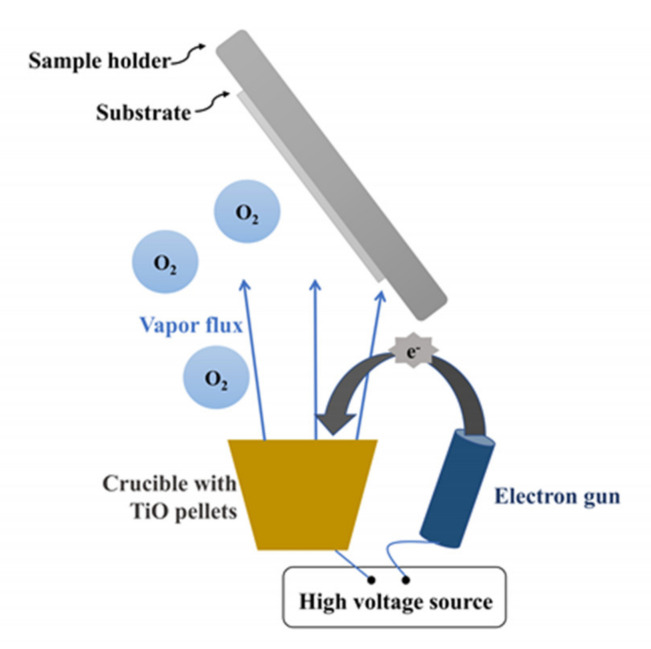
Electron-beam physical vapor deposition with OAD configuration for obtaining TiO_2_ NCs. A target anode (contained in a crucible) is bombarded with an electron beam emitted by a charged tungsten filament (electron gun) under high vacuum. The electron beam causes the evaporation of the target material: the ejected atoms react with oxygen atoms from O_2_ gas molecules and precipitate into solid form, coating the substrate with TiO_2_ nanocolumns.

TiO_2_ NCs have also been manufactured with OAD configuration but using an electron bombardment evaporator [42,43]. González-García et al. used TiO pellets as a target, 10^−4^ Torr O_2_ evaporation pressure and tilt angle between 60° and 85° for obtaining TiO_2_ nanocolumnar films (Figure 7). They found that higher deposition tilt angle induced more inclined columns and higher porosity of the films. In addition, AFM and SEM imaging studies showed that surface grain size increases with both deposition angle and film thickness.

In general, the crystalline phase and morphology of the NTOS can be controlled using the methods described above. However, it is noteworthy that the development of novel manufacturing strategies to prepare NTOS consisting of desired properties is a widely studied field, where has been shown great interest in hybrid nanoparticles for improved properties [22]. Table 1 summarizes the different morphologies and crystallinity of NTOS obtained depending on the fabrication technique.

## 3. Electrochemical Detection

**Figure 8:**
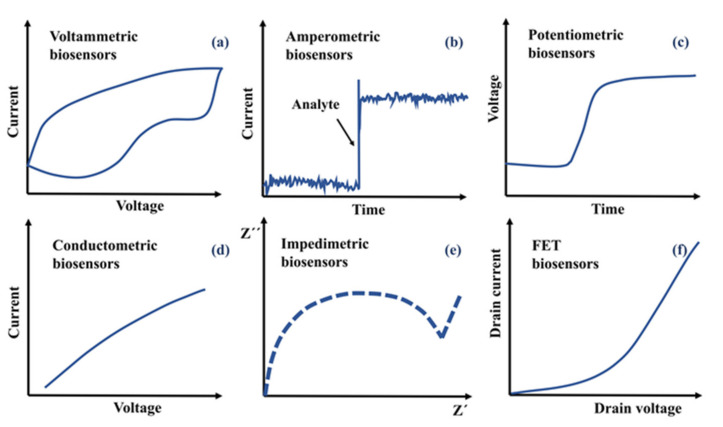
Typical signals to be measured from the different electrochemical biosensors (for any detector, support electrolyte and redox molecule used). When an analyte is detected, (**a**) the characteristic I-V curve for a voltammetric biosensor, (**b**) I-t for an amperometric biosensor, (**c**) V-t for a potentiometric biosensor, (**d**) I-V for a conductometric biosensor, (**e**) Z”-Z’ for an impedimetric biosensor or (**f**) drain current-drain voltage for a FET biosensor is obtained.

In electrochemical detection, a signal related to the analyte interaction with an electrode is measured. The measurements are carried out in different setups, including a potentiostat and two or three electrodes. Special applications, as field-effect transistors, use a little different configuration, as we discuss below. The measurements can be carried out in different ways: (a) relating the current and voltage, i.e., voltammetric and conductometric biosensors; (b) current or voltage versus time, i.e., amperometric or potentiometric, respectively; (c) imaginary versus real part of the impedance, i.e., impedimetric; and (d) drain current versus drain voltage in FET biosensors; Figure 8 schematize the signal depending on the detection mode. In this section, we describe all these configurations of electrochemical detection.

### 3.1. Voltammetric/Amperometric

Voltammetric detection is based on measuring the electric current between a working electrode (WE) and a counter-electrode (CE) by increasing and decreasing the potential at a given rate. In the amperometric setup, the current is measured versus time at a constant potential. A third electrode acts as a reference in measuring and controlling the potential of the WE, and therefore, it is called the reference electrode (RE).

The measured current depends on the oxidation or reduction reactions between the analyte and the receptor on the WE. Sensors with this operating principle detect the electrons transfer between the analyte and the WE or between a redox probe (e.g., ferricyanide ferrocene) in an electrolytic solution and the WE [4,44]. The current measurements are directly proportional to the analyte concentration at linear ranges of the potential [7]. Some parameters, such as potential (V), current (I), charge (q) and time (t), are important in this type of detection and their combined use under specific conditions gives rise to different techniques such as cyclic voltammetry (CV), linear sweep and different stripping voltammetry [4,44].

Voltammetric/Amperometric detection is the most widely used in the development of biosensors, providing a simple tool with excellent results for detection. Regarding materials, TiO_2_ NTs are used to fabricate biosensors due to their excellent biocompatibility (which allows to preserve the biomolecular nature) and high hydrophilicity (essential to immobilize biomolecules). Thus, TiO_2_ NTs are excellent substrates to “capture” or “trap” biomolecules. However, their use as electrochemical biosensors is hampered due to the low conductivity of TiO_2_ NTs.

**Figure 9:**
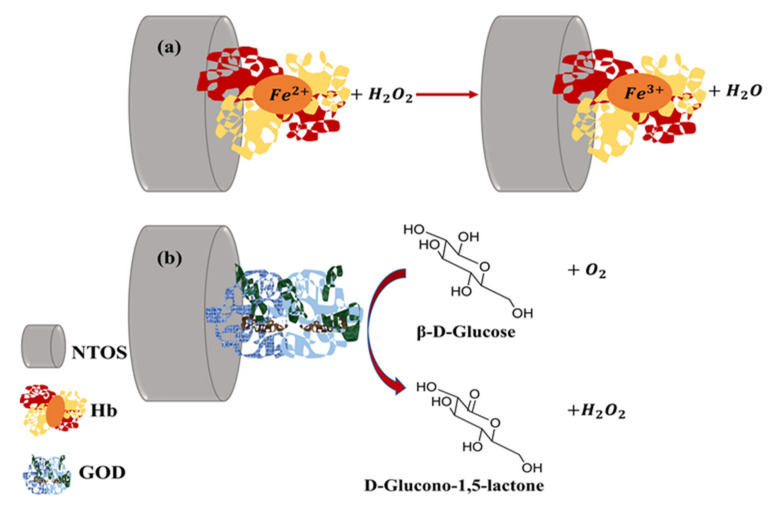
Two typical bioreceptor-analyte reactions of biosensors with a surface consisting of TiO_2_ NTs. Reduction of H_2_O_2_ catalyzed by the immobilized hemoglobin on the biosensor (**a**) and oxidation of glucose catalyzed by GOD immobilized on the biosensor (**b**).

For this reason, it is necessary to use redox mediators as in the work of Kafi et al., where they designed an H_2_O_2_ biosensor consisting of hemoglobin (Hb) modified TiO_2_ NTs onto a glassy carbon electrode, using Methylene blue (MB) as a redox mediator to enhance the electrical connection between Hb and the electrode [45]. They found that the Hb immobilized on the TiO_2_ NTs presents a high catalytic activity for the reduction of H_2_O_2_ (see Figure 9) and conserves its bioactivity despite the process used for its anchoring to the NTs. It is noteworthy that H_2_O_2_ biosensors have been extensively studied since the sensitive detection of H_2_O_2_ is important to many fields such as environmental analysis, food processing, chemical, biochemical and pharmaceutical industries.

In order to improve the conductivity of TiO_2_ NTs, carbon hybrids have been prepared. Carbon-based materials, such as amorphous ones, are among the best electrode substrates for biosensors due to their high electrical conductivity, good biocompatibility and excellent electrochemical properties. Liu et al. developed a H_2_O_2_ biosensor, without redox mediator, using carbon hybrid TiO_2_ NTs functionalized with Hb, where the crystalline phase of TiO_2_ is mainly anatase [46]. The carbon hybrid TiO_2_ NTs electrode was immersed in a 0.1 mol/L PBS (pH 6.86) containing 50 µmol/L Hb for 12 h at 4 °C to immobilize Hb. It was shown in both works (Kafie et al. and Liu et al.) that the most interesting properties of NTs were their large surface area, good biocompatibility and chemical and morphological surface properties suitable for Hb immobilization. Electrochemical experiments were realized with a three-electrode configuration, using NTs as the WE, Pt coil or foil as the CE, a KCl saturated Ag/AgCl or saturated calomel electrode as the RE, and phosphate buffer saline (PBS) as the electrolyte, in Ar or N_2_ atmosphere. In CV, after H_2_O_2_ addition to the electrochemical cell, an increase in the cathode peak is observed. The relation of the cathode peak is linear to H_2_O_2_ concentration. On the other hand, in the amperometry mode, they obtained a similar result with successive injections of H_2_O_2_ to the PBS solution in agitation, showing a linear increase in the current with the H_2_O_2_ concentration. Even though the biosensors in both papers presented rapid and sensitive responses to H_2_O_2_, those from Lui et al. showed the lowest detection limit.

Biosensors based on NTOS with the anatase phase and functionalized with Horseradish peroxidase (HRP) have been used to detect H_2_O_2_ [47,48], where chronoamperometry was used upon successive additions of H_2_O_2_ into the pH 7.0 PBS solution. For the construction of WE, Liu et al. used 12phosphotungstic acid (PTA) as a linker between Au nanoparticles (AuNPs) and TiO_2_ NTs, which also functioned as an electron mediator to accelerate electron transfer between the enzyme and the electrode. Additionally, the strong bond between HRP (previously thiolated) and AuNPs (on the biosensor surface) favored the stability and direct electron transfer of HRP on the biosensor. Furthermore, Nafion and 1-decyl-3-methylimidazolium bromide ([Demim]Br) were used to bind TiO_2_ NTs/PTA/AuNP/HRP to the surface of the glassy carbon electrode [46]. On the other hand, Guerrero et al. used a WE consisting of a glassy carbon-based electrode that was modified with carbon nanotubes (CNTs), TiO_2_ nanostructures and Prussian blue (PB) (TiO_2_ Thin film/CNTs/PB), finally functionalized with HRP, which showed a electroanalytical response towards H_2_O_2_ [47]. The results suggest that the TiO_2_ in the CNT-modified electrode improved electron transfer due to the special properties of TiO_2_. It was also demonstrated that the nanostructured environment of the film based on TiO_2_ allowed a correct immobilization of HRP and conservation of its activity. The modified electrode was shown to be stable over time, possibly due to the excellent biocompatibility of NTOS.

The presence of Ti^3+^ defects changes the electronic properties of the electrode surface based on TiO_2_ NT arrays, as it has been shown to detect H_2_O_2_ by an amperometric method [49]. Ti^3+^ defects were doped on TiO_2_ NTs arrays and then co-immobilized with HRP and thionine chloride. The electron transfer rate constant (k) was improved up to 1.34 × 10^−3^ cm/s, for annealed nanotubes in a CO atmosphere, due to Ti^3+^ defects. This result can be compared to the carbon nanotube electrode (7.53 × 10^−4^ cm/s), and the boron-doped diamond electrode (1.06 × 10^−5^ cm/s). In general, TiO_2_ NT arrays with Ti^3+^ defects had the highest amount of HRP adsorption (9.16 g/cm^2^), a faster electron transfer rate (1.34 × 10^−3^ cm/s) and the best response sensitivity. (88.5 µA/mM^−1^). Al-Fuijan et al. found that a previous electrochemical reduction process of anatase TiO_2_ nanorods allows an electrode surface modification, increasing the amount of Ti^3+^ on the NR surfaces. A physical adsorption method was implemented to immobilize the HRP enzyme on the electrode surface, where 10 µL of HRP solution was dropped onto the electrode surface and allowed to dry at RT. In addition, Nafion is used as a biosensor and as an immobilization matrix for the enzyme, which helps to maintain the stability of the biosensor. Modification of the amperometric biosensor with Ti^3+^ enhanced the detection performance of H_2_O_2_ because the electron transfer was improved [35].

Enzyme-based glucose biosensors are another widely studied biosensors due to their importance for detecting blood sugar and the control of diabetes. Glucose detection has been carried out through TiO_2_ NTs functionalized with glucose oxidase (GOD) with a fast amperometric response, high selectivity and low limit of detection (LoD) [50,51]. It could be observed in those biosensors that the response signal was sensitive to changes in glucose concentration, indicating a good electrocatalytic property of GOD functionalized electrodes (Figure 9).

Lacasse is a multicopper redox enzyme, a potential catalyst for the determination of phenolic compounds. Romero et al. fabricated an amperometric biosensor for catechol detection [52]. They prepared and deposited the composite TiO_2_/Nafion/Laccase on a graphite electrode through non-aggressive processes for the enzyme: 20 µL TiO_2_ sol (with 40 µL Nafion) and a solution of laccase (20 mg/mL), in phosphate buffer solution (pH = 6.80, 0.1 M), were mixed and sonicated; then, 23 µL of this prepared mixture were deposited on the graphite electrode. The enzymatic biosensor obtained was allowed to air-dry overnight and washed with de-ionized water before use. This process allowed to preserve the enzyme biological activity.

Lactic acid (LA) is produced in body tissues when metabolism occurs at low oxygen levels. The accumulation of LA in the body produces lactic acidosis, which is related to health disorders such as decreased tissue oxygenation, shock, left ventricular failure, sepsis, poisoning with carbon monoxide and cyanide, failure of the renal and hepatic system, diabetes and malignancy, or inborn error metabolism. In sports medicine, the level of LA in the blood is measured during exercise as an indicator for the athletic training status and fitness, where high blood levels cause a decrease in pH, leading to fatigue [53]. Yang et al. fabricated a lactic acid biosensor; for this purpose, Pt nanoparticles loaded onto TiO_2_ nanoparticles (Pt-TiO_2_ nanocomposites) were modified on the surface of a glassy carbon electrode. The electron diffraction pattern of TiO_2_ nanoparticles showed an anatase type. Subsequently, they immobilized lactate oxidase (LOx) on the surface of the sensor by dropping 5 μL of LOx solution onto Pt-TiO_2_ electrode followed by crosslinking with 5 μL of 0.1% Glutaraldehyde [54]. The amperometric detection for lactic acid in a 0.1 M PBS solution showed a linear response in the 0.003 to 0.7 mM concentration range.

In general, the most recent studies of enzyme-functionalized biosensors (with tyrosinase, urease, HRP, GOD, cytochrome c and glutamate dehydrogenase) have evidenced long-term stability, and enzyme bioactivity can be enhanced by using electrodes based on NTOS. Furthermore, it has been found that these enzymes can be immobilized on the surface or inside of the TiO_2_ nanostructure by simple processes [22].

We summarize the reviewed papers about voltammetric/amperometric detection in Table 2.

### 3.2. Potentiometric

In potentiometric detectors, the potential difference between the WE and the RE is measured: the potential difference is established due to a reaction in the cell under near-zero current flow [4]. Such potential depends on the concentration of the analyte in the solution. One of the most representative potentiometric sensors are solid-state pH detectors, which emerged to replace glass electrodes to obtain miniaturized detection systems [31]. However, other ions (F^−^, I^−^, CN−, Na^+^, K^+^, Ca^2+^, NH_4_^+^) or gas (CO_2_, NH_3_) selective electrodes are also available [5].

**Figure 10:**
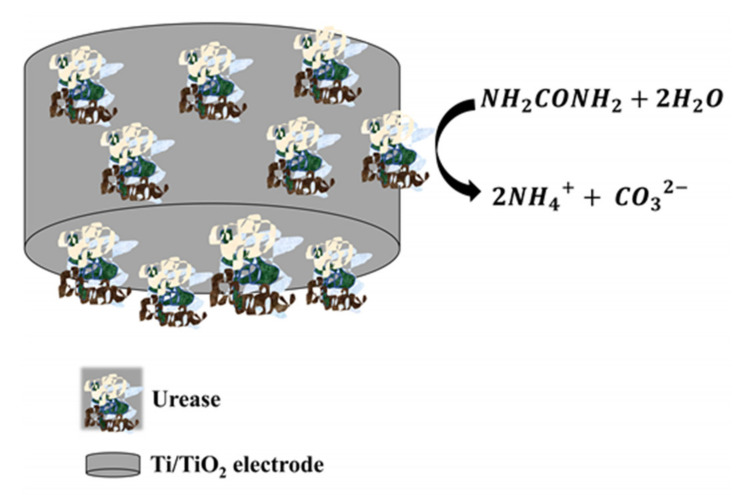
The enzymatic catalytic hydrolysis reaction in the vicinity of the urease functionalized Ti/TiO_2_ electrode.

The increase in urea concentration in pathological human fluids as blood (normal range is 15–40 mg/dL) and urine causes urinary tract obstruction, dehydration and renal failure. In contrast, the low urea levels may be related to hepatic failure, cachexia and nephritic syndrome [55]. A potentiometric urea biosensor was developed by Chen et al. using an electrochemical cell with a saturated calomel electrode, and a nanoporous TiO_2_ film electrode with immobilized urease as RE and WE, respectively [31]. These urease-imprinted thin TiO_2_ films (with anatase phase) were fabricated at the surface of Ti wires via the surface sol-gel process. The significant change in the potentiometric response of the electrode, when successive amounts of urea were added to the electrochemical cell, was due to the change in the pH of the solution associated with the urea hydrolysis reaction mediated by the urease enzyme catalyst, see Figure 10. The urea biosensor displayed a faster response time (25 s), and even its clinical testing confirmed the feasibility of detecting urea in urine samples.

### 3.3. Conductometric

The conductometric detection works by sensing changes in the conductive properties of the electrochemical system when a reaction is occurring. The configuration of a conductometric biosensor consists of two metal electrodes, a WE and a CE, for measuring the conductivity of the electrolyte layer near the WE surface. Both electrodes of this type of detector are conveniently fabricated with an interdigitated structure allowing for miniaturization [4,5].

Maniruzzaman et al. fabricated a conductometric glucose biosensor using an electrochemical cell with two electrodes, one consisting of TiO_2_ cellulose hybrid nanocomposite functionalized with glucose oxidase (onto a glass substrate) and the other consisting of a gold wire [56]. XRD pattern of this NTOS showed characteristic peaks of anatase and rutile phases of TiO_2_. For each glucose concentration (1–20 mM) used as an electrolyte solution, current vs. potential (I-V) curves were plotted. In those curves the current level increased with the increasing mass ratio of TiO_2_ in the cellulose. As detection signal, the sensitivity was used, calculated as Equation (1):

(1) $$\frac{{\left[\Delta I/\Delta V\right]}_{x}-{\left[\Delta I/\Delta V\right]}_{0}}{{\left[\Delta I/\Delta V\right]}_{0}}$$

where ${\left[\Delta I/\Delta V\right]}_{x}$ is the I-V curve slope at x mM glucose, and ${\left[\Delta I/\Delta V\right]}_{0}$ is the slope of the I-V curve at 0 mM glucose. The sensibility parameter is an indicator of enzyme activity, and it is related to the conductance. However, it should be noticed that, besides such parameters, other detection signals could be used, e.g., the conductivity at a given frequency.

### 3.4. Impedimetric

Electrochemical impedance spectroscopy (EIS) is an interfacial analytical tool, and it is widely used due to its high sensitivity with minimal hardware demand, easy production and low-cost [57]. EIS data processing produces different electrical function responses as impedance [2], admittance, complex capacitance [58], experimental chemical hardness [59] and others; in this way, EIS allows to characterize the resistive and capacitive (dielectric) properties of electrochemical systems by measuring the change in impedance when subjected to an alternating current flow [4,57]. Two setups, potentiometric and galvanometric, can measure the impedance spectrum. In the potentiometric setup, the system is perturbated by a sinusoidal voltage signal with amplitude *V*_0_, frequency f, and an appropriate offset; then, the current response, with amplitude *I*_0_, is measured; conversely, in galvanostatic configuration, a sinusoidal current with an appropriate offset excites the system, and the voltage response is measured. The impedance (Z) is defined by Equation (2):

(2) $$Z=\frac{V_{0}}{I_{0}}e^{i\varphi }$$

*φ* being the phase between current and voltage. The measurement of *Z* at different frequencies over a wide range of current intensity values is known as the impedance spectra [4,57]. As an example of other electrical function response, the complex capacitance *C^∗^* is defined by Equation (3):

(3) $$C^{*}=\frac{1}{i\omega Z}=\frac{I_{0}}{i\omega V_{0}}e^{-i\varphi }$$

where *ω* = 2πf is the angular frequency [47]. Furthermore, the measurement of the experimental chemical hardness has been proposed as the inverse of the capacitance [59,60]. The choice of the electrical function to be plotted depends on the detector response; if the behavior is mainly capacitive, the complex capacitance or the chemical hardness is the right choice, whereas the impedance should be selected in case of a main resistive response.

Typically, an impedimetric biosensor requires a redox probe, an electrolyte solution, and the use of a three-electrode cell. An example of this configuration was reported by Wang et al. where the WE consisted of TiO_2_ (Nb,V)/Chitosan nanocomposites deposited on Indium tin oxide (ITO) and then modified with DNA probe (ssDNA), the CE was a Pt electrode, the RE was an Ag/AgCl electrode and the electrolyte solution was PBS solution with a [Fe(CN)6]^3−/4−^ redox probe [61]. Here, the TiO_2_ phase, verified by XRD analysis, was rutile. An EIS characteristic curve for impedimetric biosensors is the Nyquist diagram, which is obtained by plotting the imaginary component (Z”) versus the real component (Z’) of the complex impedance (Z), see Figure 8e. The semicircular portion of the Nyquist diagram represents an electron transfer limited process. Its diameter is equal to the electron transfer resistance, R_ct_, which controls the electron transfer kinetics of the redox probe at the electrode interface. For TiO_2_-(Nb,V)/Chitosan/ITO/ssDNA biosensor in the presence of different concentrations of target DNA (a breast cancer-associated gene), the Nyquist curves showed that as the concentration of the target increased, the diameter of the semicircle also increased (R_ct_).

Halim et al. proposed another interesting example of this kind of measurement. They prepared a biosensor consisting of a Hb/TiO_2_AuNP/3Aminopropyltriethoxysilane (APTES) composite deposited on a screen-printed carbon electrode (SPCE) [62]. In this work, the properties of AuNP, such as their high conductivity and electrocatalytic behavior, are used. In addition, AuNPs also offer a higher surface area thanthat of flat gold thin film, allowing for a higher biomolecule load and thus a more sensitive biosensor. The authors obtained EIS measurements at frequencies from 100 kHz to 100 Hz in buffer solution for H_2_O_2_ detection without a redox mediator.

Karimipour et al. developed an impedimetric biosensor for prostate-specific antigen (PSA) detection. Prostate-specific antigen (PSA), a glycoprotein (serine protease), is used as a tumor marker for prostate cancer. In this way, PSA detection is useful in diagnosing prostate cancer and preventing its progression. However, PSA levels can also be elevated in benign conditions, such as benign prostatic hyperplasia. As electrode modifiers, the authors used TiO_2_-reduced Graphene oxide (rGO) hybrid nanosheets (with very thin TiO_2_ anatase nanosheets). In this biosensor, an effective synergism between GO and TiO_2_ nanosheets was exploited to immobilize aptamer molecules. Moreover, this work provides a stable aptamer-based detection model for biosensing other proteins and small molecules, given its high sensitivity (LoD = 29.4 fM) and selectivity [63].

Ognjanovic et al. deposited TiO_2_/APTES/carboxylic graphene (CG) Nanocomposites onto SPCE electrodes, by drop-casting method, for its application as a glucose biosensor [64]. They found that detecting the glucose level in a single drop of the actual sample is possible.

**Figure 11:**
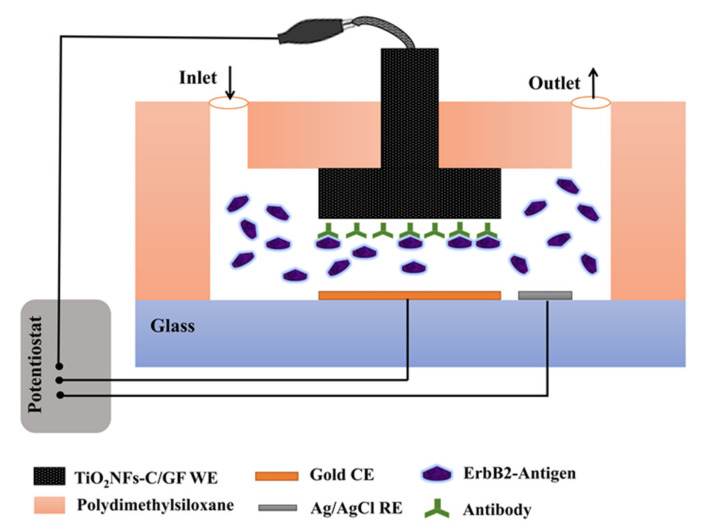
Schematic representation of the configuration of the microfluidic biosensor with 3D porous GF electrode modified with carbon-doped TiO_2_ NFs for the detection of breast cancer biomarkers.

Ali et al. used a three-dimensional WE consisting of a porous hierarchical graphite foam (GF) structure modified with carbon-doped TiO_2_ nanofibers (NFs) and functionalized with anti-ErbB2 molecules for recognition of epidermal growth factor 2 (ErbB2) [65]. The ErbB receptor family consists of four proteins: ErbB1, ErbB2, ErbB3 and ErbB4. The ErbB2 (also known as EGFR2 and HER2) is a widely studied antigen in early breast cancer diagnosis. However, the overexpression of ErbB2 may also be associated with other cancers such as ovarian, bladder, saliva, stomach and lung carcinomas. Ali et al. used a WE hung from the top of a microfluidic channel above the gold CE at the bottom of the channel. A Ag/AgCl RE was also located at the bottom of the channel and next to the counter electrode (see Figure 11). The authors were able to detect ErbB2 antigen in a selective and reproducible way by EIS measurements in PBS (pH 7.4) containing 5 mM of both [Fe(CN)_6_]^3−^ and [Fe(CN)_6_]^4−^. A miniaturized microfluidic system allowed a well-controlled microenvironment and small amounts of reagents. Furthermore, these devices could be used for multiplexed detection of mixtures of biological analytes with high performance. Table 3 summarizes NTOS-based impedimetric biosensors mentioned here.

### 3.5. Field Effect Transistor (FET)

A BioFET has two main components: a semiconducting field-effect transistor (FET, the transducer) and the biological recognition layer composed of receptors that are selective to the analyte. An insulating layer electrically separates such two elements, and the biological recognition layer is in contact with the solution to be analyzed. When the analyte binds to the receptors, the charge distribution at the surface changes and induces a change in the electrostatic surface potential of the semiconductor. Such change in the surface potential acts as a gate voltage in a traditional FET, therefore changing the current flow between the source and drain electrodes [4,15].

**Figure 12:**
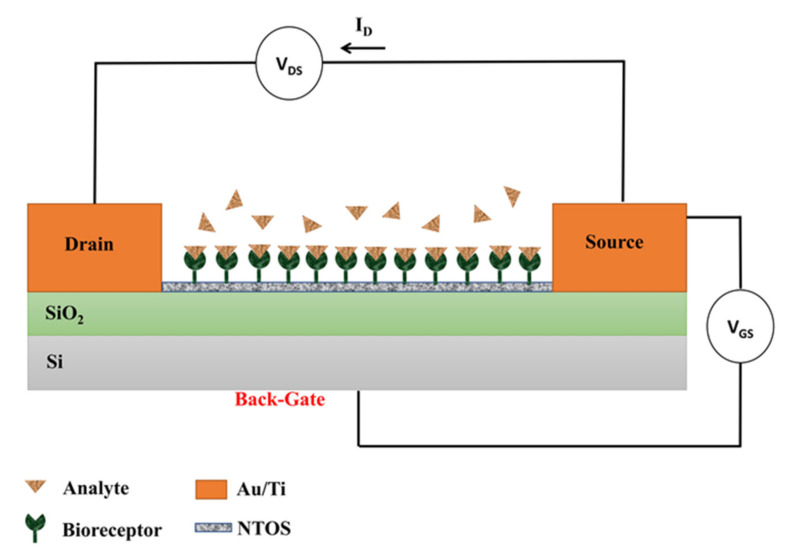
Schematic representation of the configuration of the BioFET with a back-gate.

Chu et al. studied the encapsulation of biomolecules on a nanostructured FET biosensor using polypyrrole propylic acid (PPa). PPa has a high density of carboxyl groups for covalent attach protein probes, leading to improved detection sensitivity compared to conventional immobilization methods. IgG is a generally used protein for the specific adsorption of biomolecules, so anti-rabbit IgG was used as a model, and therefore it was covalently immobilized in the PPa. PPa/anti-rabbit IgG was polymerized on the surface of anatase TiO_2_ NWs deposited onto a SiO_2_/Si substrate [66]. They exposed the active part of the biosensor to an analyte in an electrolyte solution (10 mM PBS) and measured the drain current (ID) and the potential difference between the source (S) and drain (D) channels, V_DS_, when applying different gate potentials between the gate (G) and the source (S), V_GS_ (see Figure 12). The biosensing experiments were performed at fixed V_GS_ (applied through the Si substrate): the biological recognition induced an additional back-gate voltage through the functionalized NWs that depended on the analyte concentration. The analytic curve obtained by Chu et al. showed the capability of the FET biosensor to detect low concentrations of the analyte.

Chou et al. used Ruthenium-doped rutile titanium dioxide film that was functionalized with GOD as a glucose biosensor. They observed a detection enhancement when the TiO_2_:Ru based annealed film (at 600 °C) was used as a transducer because it exhibited better nano-crystallization at the surface and therefore enhanced electrical properties. Consequently, the adsorption behavior of the enzyme immobilization and the conductive characteristics of the TiO_2_:Ru detection film are decisive variables in the reliability, repeatability, reproducibility and lifetime of the biosensor [67].

The cardiac antigen cTnI is an ideal biomarker for the detection of acute myocardial infarction (AMI) when interacting with its complementary antibody. In addition, the amount of cTnI may be associated with the severity of the AMI. Arshad et al. found that a higher sensitivity in detecting cTnI antigen could be obtained by implementing a back-gated FET biosensor to amplify the signal. For biosensor fabrication, they deposited a thin film of TiO_2_ in the channel between the source and drain regions and then functionalized it with the cTnI antibody. In this way, the device was supplied with two types of voltage, namely drain voltage (V_D_) and back-gate voltage (V_BG_), and it was shown that the characteristic I_d_–V_d_ curve increased as a function of V_BG_ [68]. In Table 4, the reviewed publications about TiO_2_ FET biosensors are summarized.

## 4. Functionalization of TiO_2_ Surfaces with Biomolecules

The functionalization of NTOS with biomolecules is a key step for the selective recognition of an analyte. Moreover, it also plays a role for the stability, since the organic coating works as a protective barrier: on the one hand, it prevents the corrosive ions from acquiring close to the surface during the electrochemical measurement [69]; on the other hand, it smooths the surface, which also helps to preserve the morphology of the nanostructured surface, since a topography with many cavities is more prone to corrosion [70].

The interactions between biomolecules and TiO_2_ surfaces can be physical or chemical, i.e., physisorption or covalent bonding processes, respectively [23]. Multi-layer deposition (disordered due to rapid reactions or interactions) or self-assembled monolayer attachment (SAMs, ordered and at thermodynamic equilibrium) are the two approaches that allow modification of the surface of substrates. The formation of multiple layers or SAMs depends on the conditions of the method used, e.g., deposition time, the type of interaction between the surface and the biomolecule (physical or chemical), and the structure of the biomolecule [71].

As it was mentioned in the introduction, metal oxides of Cu, Zn, Ni or Fe are other good surface options for biosensors, and their functionalization has also been studied [9,10,11,12]. It is important to notice that many of them share with TiO_2_ the potential for surface functionalization through the reactivity of surface bound -OH groups, which act as covalent anchoring points to various functional groups (silanes, phosphonates, carboxylates, catechols, alkenes/alkynes and amines) of specific molecules [71].

Alkylsilanes are the most widely used compounds in the preparation of monolayers on oxides due to the rapid formation of covalent bonds between the surfaces containing -OH groups and the silane anchoring groups [71]. (3-aminopropyl)triethoxysilane (APTES) is commonly used to obtain monolayers in TiO_2_ surfaces ending in amines [72], which later allow the adhesion of antibodies [68], cell receptors [73] and carboxylic graphene surface [64]. Menori et al. immobilized APTES on a TiO_2_ film by immersing the film in a 2 M solution of APTES in toluene contained in a glass bottle under a N_2_ atmosphere. The bottle was subjected to 80 °C for 2 h. Finally, the film was sonicated in toluene for 15 min and then dried with a flow of N_2_ gas. In general, this type of surface modification by alkylsilanes can be carried out employing reactions in solution (generally at RT) or vapor phase (at high temperatures, up to 120 °C). For the first approach, the viscosity of the solvent and polarity and the amount of water to hydrolyze the silane molecules are determining factors. For example, Trino et al. modified TiO_2_ thin films with (3-aminopropyl)trimethoxysilane (APTMS) by immersing the substrate in an APTMS solution for 1 min at RT [24]. In contrast, for the strategy in vapor phase, the reaction time between the silane and the surface can be several hours or days [71].

Wang et al. developed an SCC-Ag antigen biosensor consisting of a TiO_2_ coated interdigitated electrode, modified into amine by APTES to immobilize SCC-Ag antibodies attached to gold nanostars [74]. Squamous cell carcinoma antigen (SCC-Ag) is a circulating serum tumor biomarker, and high levels of this in patients have been associated with the presence of head and neck cancer. For the functionalization of the electrode, a few drops of a 3% APTES solution in ethanol were deposited on the TiO_2_ surface and kept for 3 h at RT. The surface was washed with ethanol to remove the unbound APTES residues. Gold nanostars-antibody was dropped on the surfaces and the immobilization process took place in 1 h. Then, the surface was washed with PBS buffer to complete the removal of unbound antibodies. To detect the SCC-Ag, a solution of 1M ethanolamine was used to mask the antibody-free surface areas and was kept for 30 min at RT. Next, SCC-Ag was dropped on the electrode, and the current responses before and after SCC-Ag addition were measured. A linear sweep voltage from 0 to 2 V at 0.01 V step voltage was used for the experiments. The detection limit achieved was 10 fM, while control experiments were carried out with two different proteins (serpin and albumin) that could not be recognized by anti-SCC-Ag, suggesting selective detection of SCC-Ag.

Carboxylate-metal bonding is one of the oldest approaches that have been studied to obtain SAM [71]. Carboxylic acid-modified TiO_2_ NPs can be obtained using an excellent and general solvothermal method from the solution [75]. TiO_2_ NPs in the presence of carboxylic acid (p-bromobenzoic acid) in ethanol/water (1/4 in volume) were mixed in an autoclave and heated at 100 °C for 24 h. In the work of Trino et al., 3-(4aminophenyl)propionic acid was immobilized on a TiO_2_ thin film [24]. The substrate was immersed for 5 min in an APPA solution (2 mM in ethanol) prepared previously and heated up to 40 °C.

Other reported strategies for surface modification of NTOS include the use of polymer entrapment such as polyethylene glycol (PEG) [24] or PPa [66] for immobilizing biomolecules on substrates. Venkatasubbu et al. anchored folic acid to the surface of PEG-coated TiO_2_ nanoparticles for paclitaxel transport to cancer cells [76]. The mixture of TiO_2_ NPs and PEG solution (at a mass ratio of 1:1) was agitated at 750 rpm overnight. For the binding of folic acid to PEGylated NPs, the carboxylic group of folic acid was activated using dicyclohexyl carbodiimide (DCC), and isourea was formed. On the other hand, thiols on oxides have not been intensively studied [71]. However, the formation of the S-Ti bond was studied for Trino et al. in the functionalization of TiO_2_ thin film with 3-mercaptopropionic acid, where surface characterization by XPS indicated successful functionalization [24].

**Figure 13:**
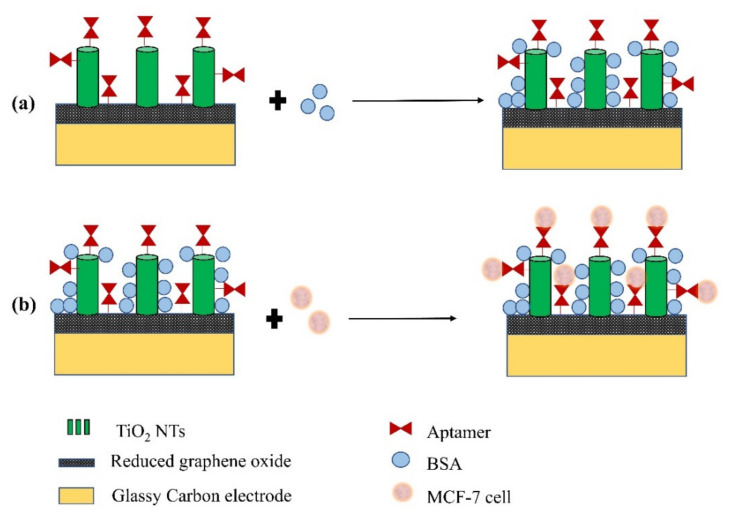
Schematic representation of the preparation of the functionalized WE for the detection of MCF-7 cells. Initially, a blockade of non-specific sites of WE is performed with BSA (**a**), and then WE are treated with the cells for detection (**b**).

Safavipour et al. developed a biosensor based on carboxylated TiO_2_ nanotubes reduced Graphene Oxide hybrids that were functionalized with MUC1 aptamer, through the interaction between the amine groups of aptamer and the carboxyl groups of hybrids, for detection of breast cancer cell (MCF-7) [77]. A pre-detection treatment with BSA solution was performed to block unspecific bindings (see Figure 13) and then wash the surface with distilled water and PBS. EIS was used to characterize the biosensor and to perform cancer cell detection, using 10 mL of PBS solution with 5mM [Fe(CN)_6_]^3−/4−^ and 0.1 mM KCl at 10 mV potential in the frequency range of 0.01 Hz–100 kHz. The selectivity of the biosensor was studied by comparing the detection results of MCF-7 cells with the detection data of the osteosarcoma cells (MG63 cell line). The detection of MCF-7 cells was successfully achieved, the detection limit being 40 cells/mL within the detection range of 10^3^–10^7^ cells/mL.

An important advantage for researchers who choose to work in this area of biosensing using NTOS is that there is a wide variety of reported studies based on NTOS, whether for sensor applications or others, that provide multiple options for both functionalization methodologies and biomolecule functionalization [78,79]. In particular, Oliveira and co-workers’ review focuses on the functionalization of TiO_2_ NTs with biomolecules for biomedical applications [25]. A wide variety of biomolecules are reported there: different peptides, epidermal growth factor, bone morphogenetic protein-2, gelatin, hemoglobin, glucose oxidase, urate oxidase, trehalose, chitosan and hyaluronic acid/hyaluronate, to name a few.

Furthermore, TiO_2_ nanomaterials have been widely explored as glucose sensors or biosensors because Ti forms coordination bonds with the carboxyl and amino groups of enzymes and conserves the enzyme activity. Rahman et al. reviewed low-temperature sol-gel and hydrothermal methods to fabricate TiO_2_ sensors or biosensors and subsequent encapsulation of enzymes [80]. Artigues et al. covalently bind GOD to TiO_2_ nanotubes coated with a tailored HEMA-co-EGDA hydrogel containing the linker, plasma-grafted pentafluorophenyl methacrylate [81]. In addition, chitosan was used as a protective agent for the enzyme. This conjugate was evaluated as an amperometric biosensor obtaining outstanding analytical results.

Finally, it is worth mentioning that Gomes et al. found different responses to the interaction between TiO_2_ films and 3-mercaptopropionic acid depending on the crystalline phase, which indicated that the functionalization in the strong acidic media needed (pH 3) occurred only for the rutile phase [82]. The non-functionalization of the anatase phase was ascribed to its oxidation during the immersion process, while the rutile phase allowed its functionalization due to the formation of the Ti-O-O-Ti dimer that reacted with that electron donor molecule [82].

## 5. Summary and Prospects

NTOS are excellent materials to fabricate electrochemical biosensors due to their large surface area, biocompatibility and stability. NTOS also have unique properties (chemical, physical and electrical) that enhance their application as transducers in biosensors.

In terms of NTOS-based biosensor fabrication, different methods allow for obtaining TiO_2_ nanostructures with the desired characteristics (shape, size, crystalline phase, roughness) that should enhance the effectiveness of the type of detection used. The heat treatment is a determining stage in most methods where amorphous TiO_2_ is obtained, and phase transitions are required for higher stability of the NTOS. Annealing temperatures between 300–500 °C are necessary to obtain the anatase phase, the preferred phase in most electrochemical biosensors, because it is insoluble in moderate acid- and alkali-based solutions and remains stable in the presence of an electrolyte and the analyte to be detected.

Another advantage of NTOS is the diversity of measurement techniques to acquire the detector response. Electrochemical biosensors are widely used due to their low cost and simplicity of the process and the fact that there is a vast understanding of electrochemical systems. Thus, it has been found that electrochemical biosensors (amperometric/voltammetric, potentiometric, conductometric, impedance, FET) based on NTOS allow the detection of analytes with high selectivity, low detection limits and short response times.

In general, to develop highly accurate, selective and repeatable NTOS-based biosensors with low detection limits, it is required to use methods to obtain NTOS in a reproducible way, to achieve a stable and robust anchorage between the bioreceptor and the NTOS, to block unspecific NTOS sites and to design biosensors that allow the amplification of the detection signal. As it has been shown in this review, there is already a good understanding of the relationship between the electrochemical behavior and the size and morphology of the NTOS. Future work is expected to integrate all these requirements in miniaturized detection systems, which would help in point-of-care medical applications.

To achieve this, the first significant challenge is to develop a quick and easy surface functionalization protocol with certain bioreceptors, for example to detect cancer. The functionalization methodology generally requires multiple steps and needs several chemicals, although in this review we have shown a few works with relatively simple functionalization processes.

Another challenging issue is the development of high selectivity in the presence of real samples, that is, under natural conditions, more complicated than those found in in vitro experiments.

Moreover, progress in the integration of microfluidics with these NTOS-based biosensors would be required. In particular, it would be quite interesting the fabrication of detection devices that could integrate different biosensing strategies (several working electrodes) to achieve multiplex detection of biological analytes in complex mixtures for high throughput experiments.

All things considered, there is still room for improvement to use these biosensors on a daily basis for the control and prevention of important socio-economic diseases such as cancer, cardiovascular problems, diabetes and COVID-19.

## Figures and Tables

**Table 1:** Relationship between NTOS type and the fabrication method.

NTOS Type	Fabrication Method	Phase	Ref.
Nanotubes	Anodization + annealing, Sol-gel + annealing	Anatase	[26,27,28,29,32]
Thin films	Sol-gel + annealing, Spray Pyrolysis, Atomic Layer Deposition, Sputtering + annealing	Anatase, rutile	[31,34,38,39]
Nanowires	Hydrothermal + annealing	Anatase and rutile mixture	[33]
Nanorods	Hydrothermal + Electrochemical reduction	Anatase	[35]
Nanocolumns	RF Sputtering, DC Sputtering + annealing, Electron-beam physical vapor deposition	Rutile, anatase	[40,41,42,43]

**Table 2:** Voltammetric/Amperometric biosensors based on NTOS.

NTOS and Additives	NTOS Fabrication Method	Bioreceptor		Analyte	LoD (µM)	Ref.
*TiO*_2_ NTs/Prussian blue/Au	Anodization	GOD		Glucose	5	[50]
*TiO*_2_ NTs/ Methylene blue /Chitosan	Anodization	Hb		H_2_O_2_	0.08	[45]
*TiO*_2_ NTs-Carbon	Anodization	Hb		H_2_O_2_	0.031	[46]
*TiO*_2_ NTs /PTA/AuNP/[Demim]Br/ Nafion	reacting polycrystalline *TiO*_2_ + NaOH solution at 110 °C for 20 h in a high pressure	HRP		H_2_O_2_	5	[48]
*TiO*_2_ NTs /bovine serum albumin/glutaraldehyde	Anodization	GOD		Glucose	3.8	[51]
*TiO*_2_ Thin film/Nafio	Sol-gel	Laccase		Catechol	0.75	[52]
*TiO*_2_ NTs-*Ti*^3+^/Nafion	Anodization + annealing in CO	HRP		H_2_O_2_	--	[49]
*TiO*_2_ NRs-*Ti*^3+^/Nafion	Hydrothermal + Electrochemical reduction	HRP	/	H_2_O_2_	0.008	[35]
*TiO*_2_ Thin film-Pt/Glutaraldehyde	photoreduction method	LOx		Lactic acid	3	[54]
*TiO*_2_ thin film /CNTs/PB	Sol-gel	HRP 17		H_2_O_2_	810	[47]

**Table 3:** Impedimetric biosensors based on NTOS.

NTOS and Additives	NTOS Fabrication Method	Bioreceptor	Analyte	LoD	Ref.
TiO_2_ NFs-C /GF	The electrospinning technique with [Ti(OiPr)_4_] was the sol-gel precursor material	Anti-ErbB2	ErbB2 antigen	1.0 fM	[65]
TiO_2_ Thin filmAuNP/APTS	Sol-gel	Hb	*H*2*O*2	10 µM	[62]
TiO_2_ Thin film-(Nb,V)/Chitosan	hydrothermal method + baking + sintering	DNA probe	Breast cancer susceptible gene	0.109 fM	[61]
TiO_2_ thin film-rGO	hydrothermal method	DNA aptamer	Prostate-specific antigen	29.4 fM	[63]
TiO_2_ Thin film/APTES/CG	Through the formation of TiO_2_ colloidal sol	GOD	Glucose	24 µM	[64]

**Table 4:** FET biosensor based on NTOS.

NTOS and Additives	NTOS Fabrication Method	Bioreceptor	Analyte	LoD	Ref.
TiO_2_ Thin film-Ru	RF sputtering + annealing	GOD	Glucose	--	[67]
TiO_2_ NWs/PPa	Hydrothermal	Anti-rabbit IgG	Rabbit IgG antigen	−3.96 A/(ng/mL) for *V_DS_* = 5 V	[64]
TiO_2_ Thin film/ APTES/ Glutaraldehyde	Sol-gel + Spin coating + annealing	Anti-cardiac troponin I	Cardiac troponin I anti gen	1 fg/mL for *V_G_* = −5 V and = 5 V	[68]

## Data Availability

Not applicable.
